# Surgical management of Warthin tumor: long-term follow-up of 224 patients from 2002 to 2018

**DOI:** 10.1007/s10006-023-01156-4

**Published:** 2023-05-16

**Authors:** Paola Bonavolontà, Cristiana Germano, Umberto Committeri, Giovanni Dell’Aversana Orabona, Pasquale Piombino, Vincenzo Abbate, Fabio Maglitto, Giorgio Iaconetta, Luigi Califano

**Affiliations:** 1 Department Neurosciences, Reproductive and Odontostomatological Sciences, Federico II University of Naples, Via pansini n. 5, 80100 Naples, Italy; 2 Department of Neurosurgery, University of Salerno, Salerno, Italy

**Keywords:** Warthin tumor, Extracapsular dissection, Pre-neural parotidectomy

## Abstract

**Purpose:**

Warthin tumors (WT) are the second most common benign parotid gland neoplasms. They can occur as synchronous or metachronous lesions in 6–10% of cases. This study aims to compare the complication rate in 224 patients who underwent extracapsular dissection (ECD) or superficial parotidectomy (SP) for the treatment of a WT.

**Methods:**

This retrospective study was conducted at the Department of Maxillo-Facial Surgery at the University of Naples “Federico II” from February 2002 to December 2018 on a group of patients who underwent surgical treatment for WT. The type of surgical technique was chosen based on Quer’s classification. The complications evaluated were facial nerve palsy, hematoma, Frey’s syndrome, and bleeding.

**Results:**

A total of 224 patients treated from 2002 to 2018 for Warthin tumor were included in the study. Two hundred elven had solitary tumors (94.1%) and 13 had multicentric lesions (5.8%), of which 9 cases presented synchronous lesions and 4 cases presented metachronous lesions. Extracapsular dissection (ECD) was performed in 130 patients (58.3% of cases) and superficial parotidectomy (SP) in the other 94 (41.7% of cases).

**Conclusions:**

We consider both surgical techniques as valid. In our opinion, it is essential to study each case based on Quer’s Classification to obtain the best surgical outcome. Based on a lower observed rate of complications such as facial nerve palsy, Frey’s syndrome, and bleeding, ECD seems to be the best option for the surgical treatment of Quer Class I lesions.

## Introduction

Although WT is the second most frequent benign tumor of the parotid gland after pleomorphic adenoma, there is a gap in the literature regarding the treatment of this neoplasm due to its particular histological features and clinical behavior [1–3]. It accounts for about 5 to 30% of benign parotid neoplasms [4]. WT is a capsulated, slow-growing tumor, rarely showing malignant transformation. In 86% of cases, it affects the parotid tail [5]. It shows a predilection for males (ratio M/F = 2.3:1) aged between the fifth and sixth decades and with a history of smoking [2, 3, 6].

Histologically, WT can be classified into three subtypes, based on the proportions of epithelial tissue and lymphoid stroma. Subtype 1, or “typical,” is formed of 50% epithelial tissue; subtype 2, or “stroma-poor,” contains 70 to 80% epithelial tissue; and subtype 3, or “stroma-rich,” is limited to only 20 to 30% epithelial tissue [7]. It is also known 64 as “cystoadenolymphoma” due to distinctive papillary structures, with cells lining cystic cavities containing eosinophilic material, lymphocytes, macrophages, and crystalloids.

**Fig. 1:**
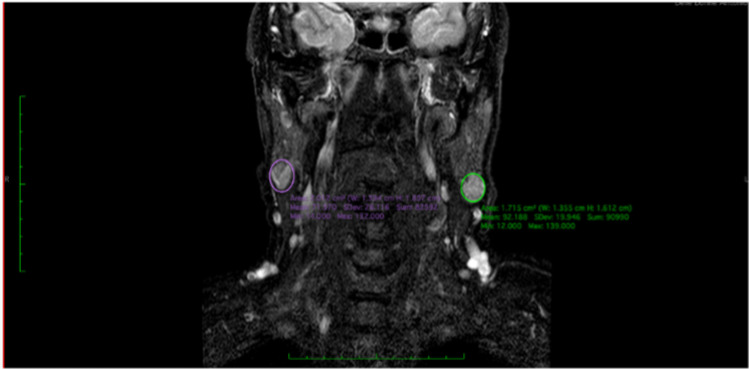
MR images of a synchronous contralateral WT in coronal projection

Ultrasound scan (US) and magnetic resonance imaging (MRI) represent the gold standard for diagnostic evaluation of WT. This lesion displays alternating solid and cystic spaces, with clear margins and soft or elastic consistency. Contralateral and ipsilateral metachronous lesions occur in about 6–10% of WT [7, 8] (Fig. 1). There is no uniformity regarding the best surgical technique for the management of WT. Superficial parotidectomy (SP) and total parotidectomy (TP) have been the most used surgical techniques for several decades [9]. Recently, extracapsular dissection (ECD) and superficial parotidectomy (SP) have been advocated as surgical treatment options for the treatment of WT.

Our study aimed to compare the rate of postoperative complications after SP and after ECD in patients treated for WT at our facility, to assess the more favorable surgical technique. Afterward, the results were evaluated considering the current literature.

## Materials and methods

A retrospective study was conducted on patients who underwent surgical treatment for parotid WT at our Department of Maxillo-Facial Surgery of the University of Naples “Federico II” from February 2002 to December 2018.

The inclusion criteria of the study were histopathological diagnosis of parotid WT and patients who underwent SP or ECD.

The exclusion criteria of the study were preoperative facial nerve dysfunction due to other causes, previous parotid gland surgeries, and non-compliance with follow-ups (in terms of incomplete records or missed appointments).

All patients underwent a thorough history and physical examination, US examination of the salivary gland, and US-guided fine needle cytology (FNAC), the latter being a key test in the diagnosis of WT. The positive predictive value (PPV) of the FNAC is approximately 86–93% [10–13]. MRI was used to complete the preoperatory assessment when WTs were close to the facial nerve branches or if the relationship between the lesion and the surrounding anatomical structures warranted further investigation.

Patients were classified following Quer’s classification [14]. Superficial, single, and mobile lesions up to a maximum of 3 cm in diameter (Quer’s Class I) underwent ECD since they can be easily identified when the gland is exposed. On the contrary, SP was performed for deep parotid lobe neoplasms (Quer’s Class II) and/or greater than 3 cm (Quer’s Classes III-IV) [15].

## Operative techniques

### ECD

The extracapsular dissection included the excision of healthy tissue from a 1.5-cm margin surrounding the lesion. A skin incision is required; normally, it is the Blair incision or facelift incision. The tumor is then identified and isolated. Care must be taken not to damage the tumor capsule or the branches of the facial nerve that might run close to it.

### SP

Superficial parotidectomy involves the removal of the superficial parotid lobe and a complete nerve dissection. The surgical technique starts with a skin incision; one of the most commonly used is the Blair incision. Then, the superficial aponeurotic muscle system is lifted, and the greater auricular nerve is identified. A major step in this technique is the identification of the common trunk of the facial nerve. The main feature of superficial parotidectomy is the dissection of the facial nerve following the course of its branches.

### Statistical analysis

Statistical calculations were done with the aid of GraphPad Prism (version 5). The statistical differences between the two techniques were analyzed using the chi-square test for discrete variables and a two-tailed Fisher test for continuous variables. Statistical significance was assessed for *p* < 0.05.

## Results

A total of 224 patients with parotid WT were included in the study. Among those, 130 patients underwent ECD and 94 patients underwent SP. The baseline characteristics of our sample are listed in Table 1. It showed a raw frequency of 92% among smokers, as seen in the literature. The mean age of patients was 62.2 years old (Graphic 1).

**Table 1:** Patient baseline characteristics. Descriptive analysis of sample baseline characteristics was performed using the Chi-squared test for discrete variables

*Anthropometric ch.*		c	
Females (%)	43 (33)	33 (35)	.82
Mean age*	61,2	63,4	.12
Smokers (%)	118 (90)	89 (94)	.82
*Topographic lesion ch.*			
Preauricular lesion (%)	36 (27)	27 (28)	.89
Inferior pole lesion (%)	94 (72)	67 (71)	.94
*Multiple lesions ch.*			
Metachronous ipsilateral (%)	2 (1)	0 (0)	.23
Metachronous contralateral (%)	1 (1)	1 (1)	.81
Synchronous ipsilateral (%)	0 (0)	6 (7)	.047
Synchronous contralateral (%)	2 (1)	1 (1)	.76
*Quer Category of lesion ch.*			
Q1 (%)	118 (90)	5 (5)	.0001
Q2 (%)	12 (9)	29 (31)	.0007
Q3 (%)	0 (0)	37 (39)	.0001
Q4 (%)	0 (0)	23 (24)	.0001

^*^Mean age of patients was analyzed using the Student’s *t*-test for continuous variables. *p* < 0.05 was considered statistically significant

**Graphic 1:**
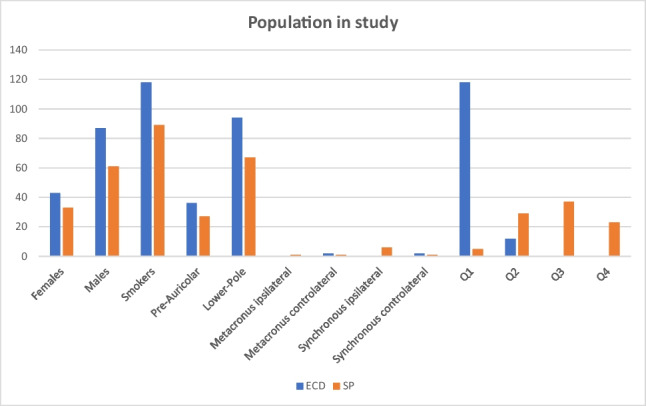
Characteristics of the study population and tumor

Most lesions (129) were located pre-auricularly and at the inferior pole. Two hundred and eleven (211) patients presented with a single lesion and 13 patients with multicentric lesions, 9 synchronous, and 4 metachronous.

The mean follow-up period was 43 months (range 24–167), and surgical complications analyzed were temporary, and permanent facial palsy, postoperative hematoma, Frey’s syndrome, and bleeding complications after SP were temporary facial palsy in 14 cases, permanent facial palsy in 4 patients, Frey’s syndrome in 6, and bleeding in 7. On the other hand, complications after ECD were 4 cases of temporary facial palsy, no cases of permanent facial palsy, 1 of Frey’s syndrome, and 2 of bleeding (Table 2). Statistical analysis showed that ECD had a significantly lower frequency of temporary facial palsy (*p* = 0.005, RR 2.61, OR 4.84), permanent facial palsy (*p* = 0.032, OR 12.43), Frey syndrome (*p* = 0.044, RR 4.06, OR 8.29), and surgical bleeding (*p* = 0.042, RR 2.61, OR 4.84). On the other hand, there was no significant statistical difference between the two described techniques for postoperative hematoma (*p* = 0.08, RR 1.88, OR 3.12).

**Table 2:** Surgical complications raw frequency

*Surgical complications*	*ECD (n. 130)*	*SP (n.94)*	*p-value*	*Significance*	*Relative risk (RR)*	*ODDS ratio (OR)*
*Temporary facial palsy (%)*	4 (3)	14 (15)	.005	Yes	2.61	4.84
*Permanent facial palsy (%)*	0 (0)	4 (4)	.032	Yes	–	12.43
*Hematoma (%)*	4 (3)	9 (9)	.08	No	1.88	3.12
*Frey syndrome (%)*	1 (1)	6 (6)	.044	Yes	4.06	8.29
*Bleeding (%)*	2 (1)	7 (7)	.042	Yes	2.61	4.84

Two-tailed Fisher exact test was used for discrete variables and adopted for the purpose of statistical inference. *p* < 0.05 was considered statistically significant

The effects of NIM use on temporary and permanent facial palsy after ECD and SP were also recorded (Table 4). Before the introduction of NIM in our facility in 2016, temporary facial nerve was encountered in 3 patients who underwent ECD, and in 12 patients who underwent SP; meanwhile, no case of permanent facial nerve palsy occurred after ECD, and 4 cases occurred after SP. The use of NIM reduced these complications to only 1 case of temporary palsy after ECD and 2 cases of temporary palsy after SP.

## Discussion

Warthin’s tumor is a capsulated, slow-growing tumor, frequently affecting middle-aged males with a history of smoking [2]*.* It accounts about 5 to 30% of benign parotid neoplasms [4], rarely showing malignant transformation, and it is located in 86% of cases in the parotid tail [5].

Due to the close anatomical relationship with vessels and nerves, surgical treatment of WT of the parotid gland can cause very disabling complications. The main complications are paralysis of the facial nerve (temporary or permanent), Frey’s syndrome, hematoma, or bleeding.

Our study aimed to compare the rate of postoperative complications after SP and ECD in patients treated for WT in our facility. Our experience was based on a large sample of 224 patients, consisting of 130 patients undergoing ECD and 94 ECD.

ECD compared to SP showed a lower incidence of temporary facial palsy (*p* = 0.005, RR 2.61, OR 4.84), permanent facial palsy (*p* = 0.032, OR 12.43), Frey syndrome (*p* = 0.044, RR 4.06, OR 8.29), and surgical bleeding (*p* = 0.042, RR 2.61, OR 4.84). While no statistically significant difference was identified between the two techniques described for postoperative hematoma (*p* = 0.08, RR 1.88, OR 3.12).

Our data are consistent with the existing literature regarding the incidence of complications (Table 3) [16–19]. In their large cohort study, Mantsopoulos et al. also demonstrated a lower incidence of permanent facial paralysis in patients undergoing ECD (1.9%) compared to SP (2.7%) [18].

**Table 3:** Studies investigating the differences of superficial parotidectomy and extracapsular dissection

Author	N SP	PFNP, %	TFNP, %	N ECD	PFNP, %	TFNP, %
Koch 2010	134	0.7	25.6	34	0	5.9
Uyar 2011	20	0	15	21	0	0
Barzan 2012	50	6	*	299	13.3	*
Orabona 2013	56	8.9	26.7	176	0	3,9
Mantsopoulos 2015	438	2,7	*	796	1.9	*
Kadletz 2016	499	0,6	10.6	395	2.2	11.4
Hoon Lee 2017	32	5	*	40	1	*

*SP* superficial parotidectomy, *PFNP* permanent facial nerve palsy, *TFNP* temporary facial nerve palsy, *ECD* extracapsular dissection

Another retrospective study consisting of 56 patients who underwent SP and 176 patients who underwent ECD demonstrated a rate of permanent facial nerve dysfunction of 8.9% after SP, whereas there were no cases after ECD [20].

Contrary to our results, Kadletz et al. showed that permanent facial palsy occurred significantly more after ECD than SP (2.2% vs 0.6%). In their study, including 395 ECDs and 499 SPs, SP was their preferred surgical technique for excision of benign parotid tumors regardless of their size and location, because it allowed better identification of the facial nerve [3]*.*

Regarding other postoperative complications, Barzan et al. in their study of 165 cases of WT supported ECD for the reduced incidence of salivary fistula (0.3% ECD vs 4% SP) and Frey’s syndrome (1.3% ECD vs 44% SP) [16]. These results were consistent with our experience. In fact, Frey’s syndrome occurred in 6% of SP-treated patients, compared to 1% of ECD-treated patients.

The preservation of more healthy glandular tissue is reported in literature as another aspect in favor of ECD [21–23]. Park et al. described 43 cases of surgically treated benign parotid tumors, demonstrating a postoperative basal salivary flow rate after ECD and SP of 0.39 and 0.14 mL/min, respectively [23].

In WT, metachronous lesions, contralateral or ipsilateral, occur in approximately 6–10% of surgically treated patients [7, 8]. SP may be the best choice to avoid subsequent parotid surgery. In the present study, we did not evaluate these lesions in the statistical analysis because they are infrequent.

We detected only 4 metachronous lesions, 2 ipsilateral and 2 contralateral, which were discovered during follow-up.

The ipsilateral metachronous lesions developed in two patients treated with ECD occurred in different parotid regions. After the first surgery, histopathology confirmed the diagnosis of WT with free margins. These two patients underwent SP after the appearance of the ipsilateral metachronous lesion. Instead, the two patients with contralateral lesions underwent ECD.

Also in a study by Lee et al. consisting of 78 patients who underwent ECD or SP, there was only one case of a metachronous lesion in a patient who had previously undergone ECD [17]. Mantsopoulos et al. also showed that only 3.1% of 197 patients undergoing ECD developed metachronous tumors [24].

The first parotidectomy with facial nerve monitoring was described in 1990 [25]. In recent decades, the use of NIM has increased significantly to prevent facial nerve damage [25].

Sood et al. demonstrated that the incidence of post-surgical facial nerve deficit was reduced by the use of NIM with both procedures, ECD and SP [26].

After its introduction at our facility in 2016, we observed a 67% reduction in temporary facial nerve palsy following ECD, an 83% reduction in temporary facial nerve palsy following SP, and permanent palsy did not occur at all in both procedures (Table 4).

**Table 4:** Comparison between cases of temporary and permanent facial nerve paralysis before and after NIM introduction in our department

Time line	Total cases	Extracapsula dissection (ECD) Vs superficial parotidectomy (SP)	Permanent palsy (PP) Vs temporary palsy (TP)
2002–2016	178 without N.IM	102 ECD	0 P
			3 T
		76 SP	4 P
			12 T
2016–2018	46 with N.I.M	28 ECD	0 P
			1 T
		18 SP	0 P
			2 T

Contrary to our results, Graciano et al. reported that the incidence of facial nerve damage remained unchanged with the use of NIM [27].

A limitation of the study was that patients were not operated by the same surgeon. This could represent a bias, especially with regard to complications. Another limitation is that a multicenter study would be needed to obtain more scientific evidence.

In conclusion, in our opinion, both surgical techniques should be considered valid. To achieve the best results, it is essential to study each case in detail.

From the evaluation of our data, ECD was indicated for superficial, mobile, single lesions with a maximum diameter of 3 cm, localized near the parotid edges (Quer’s class I).

In our experience, the ECD has superior clinical results and appears to reduce the number of complications, ensuring a more rapid post-operative course.
